# Performance-based comparison of Yamada–Ota and Hamilton–Crosser hybrid nanofluid flow models with magnetic dipole impact past a stretched surface

**DOI:** 10.1038/s41598-021-04019-8

**Published:** 2022-01-07

**Authors:** Hina Gul, Muhammad Ramzan, Kottakkaran Sooppy Nisar, Roshan Noor Mohamed, Hassan Ali S. Ghazwani

**Affiliations:** 1 Department of Computer Science, Bahria University, Islamabad, 44000 Pakistan; 2 Department of Mathematics, College of Arts and Sciences, Prince Sattam bin Abdulaziz University, Wadi Aldawaser, 11991 Saudi Arabia; 3 Department of Pediatric Dentistry, Faculty of Dentistry, Taif University, PO Box 11099, Taif, 21944 Saudi Arabia; 4 Department of Mechanical Engineering, Faculty of Engineering, Jazan University, Jazan, 45124 Saudi Arabia

**Keywords:** Software, Mechanical engineering

## Abstract

The nanofluid flows play a vital role in many engineering processes owing to their notable industrial usage and excessive heat transfer abilities. Lately, an advanced form of nanofluids namely “hybrid nanofluids” has swapped the usual nanofluid flows to further augment the heat transfer capabilities. The objective of this envisaged model is to compare the performance of two renowned hybrid nanofluid models namely Hamilton–Crosser and Yamada–Ota. The hybrid nanoliquid (*TiO*_2_-*SiC/DO*) flow model is comprised of Titanium oxide (*TiO*_2_) and Silicon carbide (*SiC*) nanoparticles submerged into Diathermic oil (*DO*). The subject flow is considered over a stretched surface and is influenced by the magnetic dipole. The uniqueness of the fluid model is augmented by considering the modified Fourier law instead of the traditional Fourier law and slip conditions at the boundary. By applying the suitable similarity transformations, the system of ordinary differential equations obtained from the leading partial differential equations is handled by the MATLAB solver bvp4c package to determine the numerical solution. It is divulged that the Yamada–Ota model performs considerably better than the Hamilton–Crosser flow model as far as heat transfer capabilities are concerned. Further, the velocity reduces on increasing hydrodynamic interaction and slip parameters. It is also noted that both temperature profiles increase for higher hydrodynamic interaction and viscous dissipation parameters. The envisioned model is authenticated when compared with an already published result in a limiting case.

## Introduction

The name “Magnetohydrodynamics” was introduced by the Swedish national Nobel Laureate Hannes Alfven. The role of magnetohydrodynamics is significant in fluid dynamics and possesses several industrial applications including metallurgy, crystal growth, polymer technology, MHD accelerators, fiber production, and plastic extrusion, etc. Takhar et al.^1^ revealed that the surface drag coefficient is considerably increased when a magnetic field is applied, although the heat transfer rate is slightly reduced. The magnetic impact on *CuO*-*H*_2_*O* nanofluid is studied by Sheikholeslami et al.^2^ using the Lattice Boltzmann and Koo–Kleinstreuer–Li correlation methods. It is concluded in this study that for large Rayleigh number, the influence of heat source length and Hartmann number is boosted. Seth et al.^3^ deliberated the impacts of MHD, viscous dissipation, Joule heating, and non-Darcy Casson fluid near a vertical extended plate through a permeable medium. The finite difference implicit approach of the Crank–Nicolson type is used to get numerical results. Ramzan et al.^4^ considered Oldroyd-B ferromagnetic nanofluid flow with magnetic dipole over an extended stretching sheet. Some recent applications for magnetic dipole may be found in Refs.^5–9^.

In the modern era of technological advancement, hybrid nanofluids, a modified class of nanofluids, have been introduced. Nanofluids are made up of single-type metal nanoparticles inserted into the customary fluid nevertheless hybrid nanofluids are made-up of two or more metallic nanoparticles addition into the base liquid. Hybrid nanofluids have drawn the attention of scientists and researchers to look for more possibilities in this novel research area owing to their improved thermal conductivity and heat transfer capabilities than the common nanofluids. Chung et al.^10^ deliberated the effects of hybrid nanoliquid with Copper and Graphene Oxide nanoparticles with base fluid engine oil in a partially ionized flow. The flow is taken on a surface that is extended in a nonlinear way. It is observed that the high collision rate of ions and electrons triggers the hybrid magnetized nanoliquid flow. The Ion slip and Hall current are produced by this collision rate. The fluid is subjected to a force that is the opposite of the magnetic force. The new idea about hybrid nanofluid with Yamada–Ota and Xue model with a surface catalyzed reaction that improves the reaction rate is discussed by Riasat et al.^11^. From this investigation, it is concluded that axial velocity delines in the case of gases but boosts in the case of liquids. Nayak et al.^12^ used the Hamilton–Crosser model to assess the shape influences and interfacial layer of carbon nanotubes (CNTs) on water nanofluid flow between two extendable disks. The irreversibility analysis of the problem is also evaluated. It is understood that flow is strengthened along the axial and radial directions owing to enriched nanoparticles’ shape factor and a reverse effect is witnessed along the tangential direction. The flow of an immersed nanoparticles hybrid nanofluid with viscosity and variable thermal conductivity are studied by Abbas et al.^13^. With pure water as the basis fluid, two types of nanoparticles, MWCNTs, and SWCNTs are added to form the hybrid nanofluid. Abbas et al.^14^ also studied the Micropolar hybrid nanofluid considering Yamada–Ota and Xue model over a permeable curved exponentially expanding channel surface. It is perceived that the rate of heat transfer is greater for the Yamada–Ota models of hybrid nanoliquid than the Xue model. Some recent hybrid nanofluids studies are given at^15–18^.

The transmission of heat is a studied phenomenon that occurs when temperature variations exist between two distinct objects or inside the same body. According to the Fourier law (heat conduction), any disturbance that occurs at the beginning will continue throughout the process. To overcome the problem, Cattaneo included a thermal relaxation period in Fourier’s law (heat conduction), allowing heat to be transported by waves propagating at a controlled speed^19–21^. Later, using Oldroyd’s upper-convected derivative and frame-indifferent change, Christov developed the Cattaneo relation. The Cattaneo–Christov (C–C) flux model is named after this relationship. Over distinct geometries (wedge, plate, and cone), Makinde et al.^22^ investigated the cumulative influence of the external magnetic field, C–C heat flux, buoyancy forces, heat source, and chemical reaction on the movement of an incompressible liquid electrically conducting with mass and heat transfer. Gireesha et al.^23^ studied the characteristics of melting heat transfer and MHD flow and of dusty Casson fluid with C–C heat flux past over a stretching layer.

The above-cited references and the available literature signify that the presented model is unique and has not been studied in the literature yet. The goal of this novel study is to compare the performance of Hamilton–Crosser and Yamada–Ota magnetic dipole hybrid nanofluid flow that is based on Titanium oxide and Silicon carbide (*TiO*_2_-*SiC*) nanoparticles with base fluid Diathermic oil (*DO*) on an extended sheet with Cattaneo–Christov (C–C) heat flux and partial slip condition at the boundary of the surface. Comparison of both the thermal conductivity models (Hamilton–Crosser, and Yamada–Ota) are presented considering different physical parameters. For both types of models, the Skin friction coefficient effects are also included. The MATLAB solver bvp4c is employed to solve the complex nonlinear equations. The behavior of the related parameters is discussed using graphical findings. Table 1 is presented to distinguish the present work from the published literature.

**Table 1:** Comparison of the current work with the closely related published papers.

References	C–C heat flux	Slip boundary condition	(*TiO*_2_-*SiC/DO*)	Hybrid nanofluid	Yamada–Ota model	Hamilton–Crosser model
^9^	No	No	No	Yes	No	Yes
^24^	No	No	No	Yes	No	No
^25^	No	No	No	Yes	No	No
^26^	No	No	No	Yes	No	No
Present	Yes	Yes	Yes	Yes	Yes	Yes

It is comprehended from Table 1 that the envisioned model is novel and no such idea is discussed in the literature yet.

## Mathematical formulation

The mathematical model is erected considering the subsequent assumptions:

i. The fluid flow is incompressible.
ii. The flow is under the magnetic dipole effect.
iii. The fluid flows in a positive *x-*axis direction from left to right.
iv. The fluid is flowing with the velocity $U_{w} = Sx,$ where $S$ is a stretching constant.
v. The distance between the surface and the magnetic dipole centered at the *y-*axis is taken as C.
vi. Hamilton–Crosser and the other is Yamada–Ota hybrid models are compared.
vii. Silicon carbide and Titanium oxide nanoparticles are immersed into Diathermic oil.
viii. The flow is under the influence of Cattaneo–Christov heat flux.
ix. The partial slip boundary condition is also considered.

**Figure 1:**
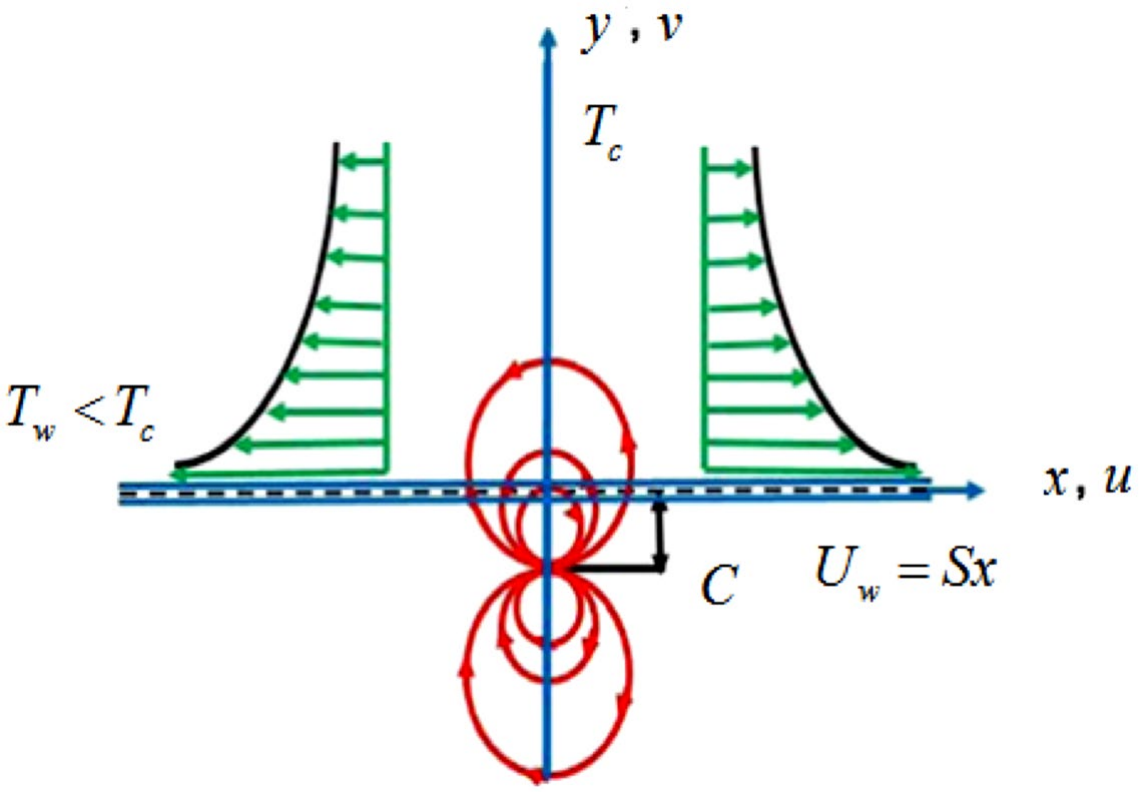
Schematic flow diagram.

Figure 1 is drawn to show the magnetic dipole effect and the flow pattern.

The model equations are expressed considering the above assumptions^4,9,27^:

1 $$u_{x} + v_{y} = 0,$$

2 $$\rho_{HNF} \left( {uu_{x} + vu_{y} } \right) = \mu_{HNF} \left( {u_{yy} } \right) + \frac{{\mu_{0} M}}{{\rho_{HNF} }}H_{x} ,$$

3 $$\begin{aligned} uT_{x} + vT_{y} = & \frac{{k_{HNF} }}{{(\rho c_{p} )_{HNF} }}\left( {T_{xx} + T_{yy} } \right) - \lambda_{2} \left( \begin{gathered} u^{2} T_{xx} + uu_{x} T_{x} + uv_{x} T_{y} \hfill \\ + 2uvT_{xy} + vv_{y} T_{y} + vu_{y} T_{x} + v^{2} T_{yy} \hfill \\ \end{gathered} \right) \\ & - \frac{{\mu_{f} }}{{(\rho c_{p} )_{HNF} }}TM_{T} (uH_{x} + vH_{y} ), \\ \end{aligned}$$

with a set of subsequent conditions:

4 $$\begin{gathered} u = U_{w} + \mu L_{1} u_{y} ,\,\,\,\,v = 0,\,\,\,T = T_{w} ,\,\,\,\,{\text{at}}\,\,\,\,y = 0, \hfill \\ u \to 0,T \to T_{\infty } \,\,\,{\text{as}}\,\,\,\,y \to \infty . \hfill \\ \end{gathered}$$

### Magnetic dipole

Once a magnetic field is introduced, the flow of nanoliquid across the spreading sheet is affected. The resulting in a magnetic field region denoted by $\delta_{1}$ and is given by:

5 $$\delta_{1} = \frac{{\gamma_{1} }}{2\pi }\frac{x}{{x^{2} + (y + c)^{2} }}.$$

In the preceding equation, $\gamma_{1}$ denotes the magnetic field’s strong point at the base, whereas c denotes the magnetic dipole's displacement. The mathematical components of the magnetic field (H) are as follows:

6 $$H_{x} = - \frac{{\partial \delta_{1} }}{\partial x} = \frac{{\gamma_{1} }}{2\pi }\frac{{x^{2} - (c + y)^{2} }}{{\left( {x^{2} + (c + y)^{2} } \right)^{2} }},$$

7 $$H_{y} = - \frac{{\partial \delta_{1} }}{\partial y} = \frac{{\gamma_{1} }}{2\pi }\frac{2x(c + y)}{{\left( {x^{2} + (c + y)^{2} } \right)^{2} }}.$$

After differentiating Eq. (5) for magnetic field components, we get the Eqs. (6) and (7) w.r.t *x* and *y*. Because magnetic force has a direct relationship with gradient (H), analytically expresses as:

8 $$H = \sqrt {\left( {\frac{{\partial \delta_{1} }}{\partial x}} \right)^{2} + \left( {\frac{{\partial \delta_{1} }}{\partial y}} \right)^{2} } .$$

We got the following equations by putting the values into the preceding equation.

9 $$H_{x} = \frac{{\gamma_{1} }}{2\pi }\frac{2x}{{(y + c)^{4} }},$$

10 $$H_{y} = \frac{{\gamma_{1} }}{2\pi }\left( {\frac{ - 2}{{(y + c)^{3} }} + \frac{{4x^{2} }}{{(y + c)^{5} }}} \right).$$

Because temperature variations might cause changes in magnetization, the effects on magnetization can be mathematically be represented as:

11 $$M = (T_{c} - T)\;{\text{K}}.$$

Hybrid nanoparticles $TiO_{2} { - }SiC/Do$ thermo-physical properties are displayed in Table 2.

**Table 2:** Thermophysical properties of $TiO_{2} { - }SiC/DO$^28^.

Physical properties	$DO$	$SiC$	$TiO_{2}$
$\rho \left( {\frac{{{\text{kg}}}}{{{\text{m}}^{3} }}} \right)$	855	3370	4230
$c_{p} \left( {\frac{{\text{J}}}{{\text{kg K}}}} \right)$	2030	1340	692
$k\left( {\frac{{\text{W}}}{{\text{m K}}}} \right)$	0.133	150	8.4

Transformation are^29–31^:

12 $$\,\psi (\xi ,\eta ) = S\xi \nu_{F} F(\eta ),\,\,(\xi ,\eta ) = \left( {\sqrt {\frac{{\rho_{F} S}}{{\mu_{F} }}} x,\sqrt {\frac{{\rho_{F} S}}{{\mu_{F} }}} y} \right).$$

in which ψ is the stream function and $(\xi ,\eta )$ are the dimensionless parameters. The expression for the thermal and solutal distribution along with velocity components are given as

13 $$\begin{gathered} u = \frac{\partial \psi }{{\partial y}} = SxF^{\prime}(\eta ),v = - \frac{\partial \psi }{{\partial x}} = - \sqrt {S\nu_{F} } F(\eta ),\theta (\eta ) = \frac{{T_{c} - T}}{{T_{c} - T_{w} }}. \hfill \\ \,\,\,\,\,\,\,\,\,\,\,\,\,\,\,\,\,\,\,\,\,\,\,\,\,\,\,\,\,\, \hfill \\ \end{gathered}$$

The model Eqs. (1)–(4) take the form:

14 $$\frac{1}{{A_{1} \rho_{HNF} }}F^{\prime\prime\prime} - F^{^{\prime}2} + FF^{\prime\prime} - \frac{{2\beta \theta \left( {\eta + \alpha } \right)^{4} }}{{\rho_{HNF} }} = 0,$$

15 $$\frac{{A_{2} }}{{(\rho c_{p} )_{{HNF}} }}\theta ^{\prime\prime} + \Pr \left[ {F\theta ^{\prime} - 2F^{\prime}\theta - \gamma \left( {F^{2} \theta ^{\prime\prime} + FF^{\prime}\theta ^{\prime}} \right)} \right] + \frac{{2\beta \lambda \left( {\theta - \varepsilon } \right)}}{{\left( {\eta + \alpha } \right)^{3} \rho _{{HNF}} }}F - 4\lambda F^{{'2}} = 0,$$

16 $$\begin{array}{*{20}l} {F = 0,\;F^{\prime} = 1 + \delta F^{\prime\prime},\;\theta (\eta ) = 1} \hfill & {{\text{at}}\;\eta = 0} \hfill \\ {F^{\prime} \to 0,\;\theta \to 0} \hfill & {{\text{as}}\;\eta \to \infty } \hfill \\ \end{array} .$$

The non-dimensional variables are defined as:

17 $$\begin{gathered} \delta = \rho_{F} L_{1} \sqrt {\nu_{F} S} ,\,\,\,\beta = \frac{\gamma }{{2\pi \mu_{F}^{2} }}\mu_{0} k_{F} \frac{{\left( {T_{c} - T_{w} } \right)}}{{\mu_{F}^{2} }}\rho_{f} ,\Pr = \frac{{\nu_{F} }}{{\alpha_{F} }},\varepsilon = \frac{{T_{c} }}{{T_{c} - T_{w} }}, \hfill \\ \lambda = \frac{{S\mu_{F}^{2} }}{{\rho_{F} k_{F} (T_{c} - T_{w} )}},\alpha = \sqrt {\frac{{S\rho_{F} c^{2} }}{{\mu_{F} }}} ,\gamma = \lambda_{1} S. \hfill \\ \end{gathered}$$

## Physical quantities

The coefficient of Skin friction of the hybrid nanofluid on the surface can be calculated as:

18 $$\,C_{F} = \frac{{\mu_{HNF} \left. {u_{y} } \right|_{y = 0} }}{{\rho_{HNF} U_{w}^{2} }},\,$$

The nondimensional expression of $C_{f}$ is written as:

19 $$\,C_{F} {\text{Re}}_{x}^{1/2} = \frac{{A_{1} F^{\prime\prime}(0)}}{{A_{0} }}.$$

with ${\text{Re}}_{x}^{1/2} = \frac{{xU_{w} (x)}}{{\upsilon_{f} }} = \frac{{Sx^{2} }}{{\nu_{F} }}$ as local Reynold number.

Two thermal conductivity models, namely Hamilton Crosser and the Yamada-Ota models are utilized in this study to analyze the thermal properties of nanofluid. Table 3 depicts the thermophysical traits of the hybrid nanofluid flow of Yamada-Ota and Hamilton Crosser models.

**Table 3:** Thermophysical properties of Hybrid nanoliquid^32^.

Density	$\rho_{HNF} = \rho_{F} \left( {1 - \varphi_{2} } \right)\left( {(1 - \varphi_{1} ) + \varphi_{1} \left( {\frac{{\rho_{p1} }}{{\rho_{F} }}} \right)} \right) + \varphi_{2} \rho_{{p_{2} }} ,\frac{{\rho_{HNF} }}{{\rho_{F} }} = A_{0}$
Heat capacity	$$\begin{gathered} (\rho c_{p} )_{HNF} = \varphi_{2} (\rho c_{p} )_{{p_{2} }} + (1 - \varphi_{2} )(\rho c_{p} )_{F} \left\{ {\varphi_{1} } \right.\frac{{(\rho c_{p} )_{{p_{1} }} }}{{(\rho c_{p} )_{F} }} + [(1 - \varphi_{1} )\left. ] \right\} \hfill \\ \hfill \\ \end{gathered}$$
Variable viscosity	$\mu_{HNF} = \frac{{\mu_{F} }}{{(1 - \varphi_{1} )^{2.5} (1 - \varphi_{2} )^{2.5} }},\,\,\,\,\,\frac{{\mu_{HNF} }}{{\mu_{F} }} = A_{1}$
Thermal conductivity	$$\begin{gathered} \frac{{k_{{HNF}} }}{{k_{{bF}} }} = \frac{{k_{{p_{2} }} - k_{{bF}} (1 - n) - (k_{{p_{2} }} - k_{{bF}} )(1 - n)\varphi _{2} }}{{(n - 1)k_{{bF}} + k_{{p_{2} }} - (k_{{p_{2} }} - k_{{bF}} )\varphi _{2} }} \hfill \\ \frac{{k_{{bF}} }}{{k_{F} }} = \frac{{k_{F} (n - 1) + k_{{p_{1} }} + (k_{F} - k_{{p_{1} }} )(1 - n)\varphi _{1} }}{{(n - 1)k_{F} + k_{{p_{1} }} - (k_{{p_{1} }} - k_{F} )\varphi _{1} }},\,\frac{{k_{{HNF}} }}{{k_{F} }} = A_{2} \hfill \\ \end{gathered}$$
Hamilton and Crosser model	$\frac{{k_{HNF} }}{{k_{bF} }} = \frac{{k_{{p_{2} }} + k_{bF} (n - 1) - (1 - n)\varphi_{2} \left( {k_{{p_{2} }} - k_{bF} } \right)}}{{k_{{p_{2} }} + (n - 1)k_{bF} + \varphi_{2} \left( {k_{bF} - k_{{p_{2} }} } \right)}}$ $\frac{{k_{bF} }}{{k_{F} }} = \frac{{k_{{p_{1} }} + k_{F} (n - 1) - (n - 1)\varphi_{1} \left( {k_{F} - k_{{p_{1} }} } \right)}}{{k_{{p_{1} }} + (n - 1)k_{F} - \varphi_{1} \left( {k_{{p_{1} }} - k_{F} } \right)}}$
Yamada–Ota model	$$\frac{{k_{HNF} }}{{k_{bF} }} = \frac{{\frac{{k_{{p_{2} }} }}{{k_{bF} }} + \psi + \psi \varphi_{2} \left( {1 - \frac{{k_{{p_{2} }} }}{{k_{bF} }}} \right)}}{{\frac{{k_{{p_{2} }} }}{{k_{bF} }} + \psi + \varphi_{2} \left( {1 - \frac{{k_{{p_{2} }} }}{{k_{bF} }}} \right)}},\,\,\left\{ \begin{gathered} \psi = 2\varphi_{2}^{0.2} \frac{L}{D}\,\,{\text{for}}\,{\text{cylindrical}}\,{\text{particle}} \hfill \\ \psi = 2\varphi_{2}^{0.2} \,\,\,\,\,{\text{for}}\,{\text{spherical}}\,{\text{particle}} \hfill \\ \end{gathered} \right.$$ $$\frac{{k_{bF} }}{{k_{F} }} = \frac{{\frac{{k_{{p_{1} }} }}{{k_{F} }} + \psi + \psi \varphi_{1} \left( {1 - \frac{{k_{{p_{1} }} }}{{k_{F} }}} \right)}}{{\frac{{k_{{p_{1} }} }}{{k_{F} }} + \psi + \varphi_{1} \left( {1 - \frac{{k_{{p_{1} }} }}{{k_{F} }}} \right)}},\,\,\,\,\,\,\left\{ \begin{gathered} \psi = 2\varphi_{1}^{0.2} \frac{L}{D}\,\,{\text{for}}\,{\text{cylindrical}}\,{\text{particle}} \hfill \\ \psi = 2\varphi_{1}^{0.2} \,\,\,\,\,{\text{for}}\,{\text{spherical}}\,{\text{particle}} \hfill \\ \end{gathered} \right.$$

## Numerical scheme

For the system of Eqs. (14)–(16), the numerical scheme of MATLAB bvp4c is applied in order to solve the ODEs. For this purpose, the first step is to establish the new variables as under:

20 $$\begin{gathered} F(\eta ) = y_{1} ,\,\,\,\theta_{1} (\eta ) = y_{4} ,\,\,\,\theta_{2} (\eta ) = y_{6} ,\,\,\,F^{\prime}(\eta ) = y_{2} ,\,\,\,\theta^{\prime}_{1} (\eta ) = y_{5} ,\,\,\,\theta^{\prime}_{2} = y_{7} , \hfill \\ F^{\prime\prime}(\eta ) = y_{3} ,\,\,F^{\prime\prime\prime}(\eta ) = yy_{1} ,\,\,\theta^{\prime\prime}_{1} (\eta ) = yy_{2} ,\,\,\,\theta^{\prime\prime}_{2} (\eta ) = yy_{3} . \hfill \\ \end{gathered}$$

Inserting the above variables in the MATLAB bvp4c, Eqs. (14) and (15) take the form of first-order equations as:

21 $$yy_{1} = A_{1} \rho_{HNF} \left( {y_{2}^{2} - y_{1} y_{3} + \frac{{2\beta y_{4} (\eta + \alpha )^{4} }}{{\rho_{HNF} }}} \right),$$

22 $$yy_{2} = \frac{{\left( {\rho c_{p} } \right)_{HNF} }}{{A_{2} }}\frac{{\left( \begin{gathered} \left( { - \Pr \left[ {y_{1} y_{5} - 2y_{2} y_{4} - \gamma (y_{1} y_{2} y_{5} )} \right]} \right) \hfill \\ - 2\frac{{\beta \lambda \left( {y_{4} - \varepsilon } \right)}}{{(\eta + \alpha )^{3} }} + 4\lambda y_{2}^{2} \hfill \\ \end{gathered} \right)}}{{(1 - \gamma y_{1}^{2} \frac{{\left( {\rho c_{p} } \right)_{HNF} }}{{A_{2} }}}}.$$

And BCs in Eq. (16) shaped into the following form:

23 $$\begin{array}{*{20}c} {y_{1} (0) = 0,\,\,y_{2} (0) - 1 - \delta y_{3} ,y_{4} (0) - 1} \\ {y_{2} (\infty ),\,\,y_{4} (\infty )} \\ \end{array} .$$

**Figure 2:**
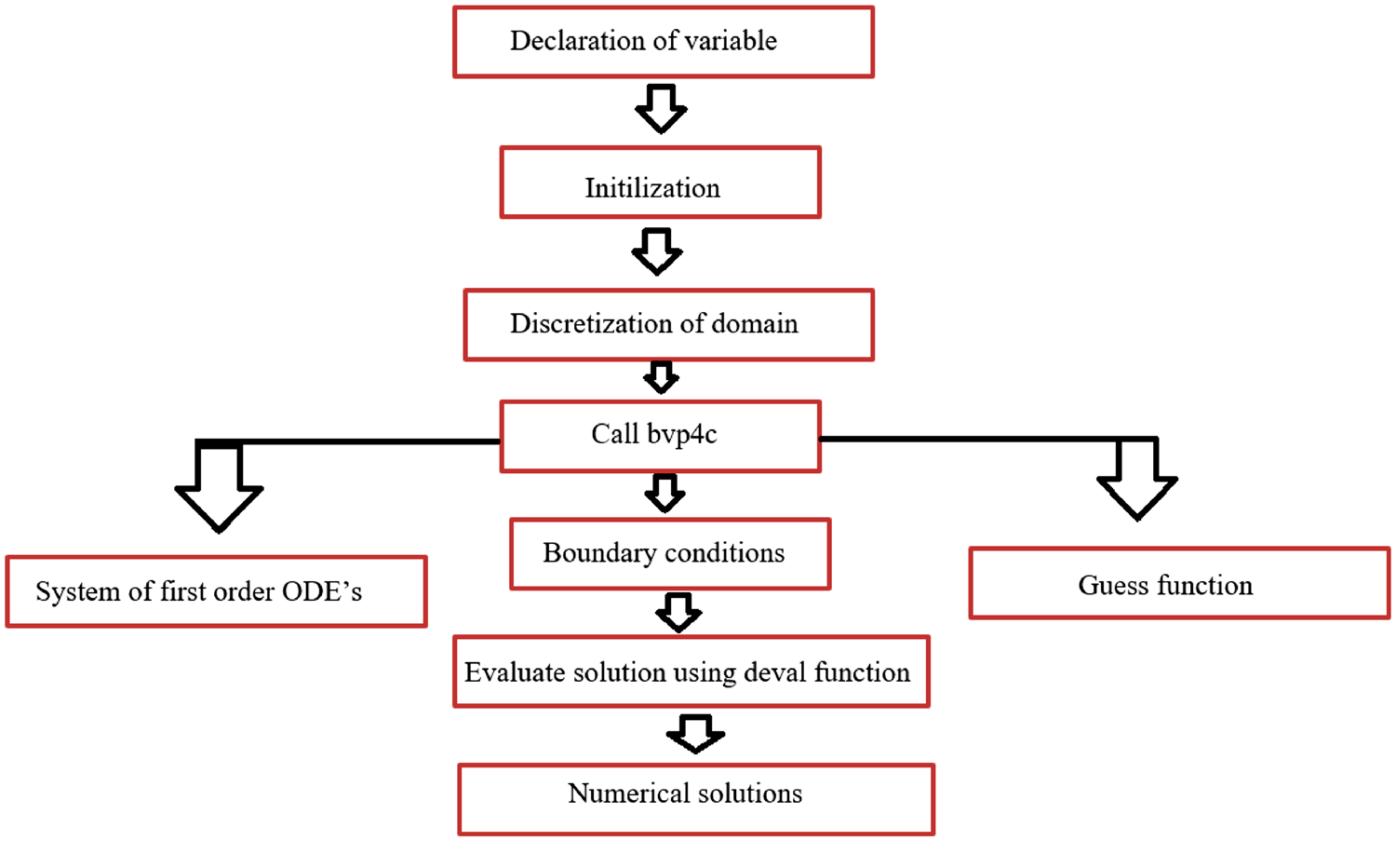
Flow plan of numerical program.

Flow chart of the numerical scheme (bvp4c) is given in Fig. 2.

## Results with discussion

**Figure 3:**
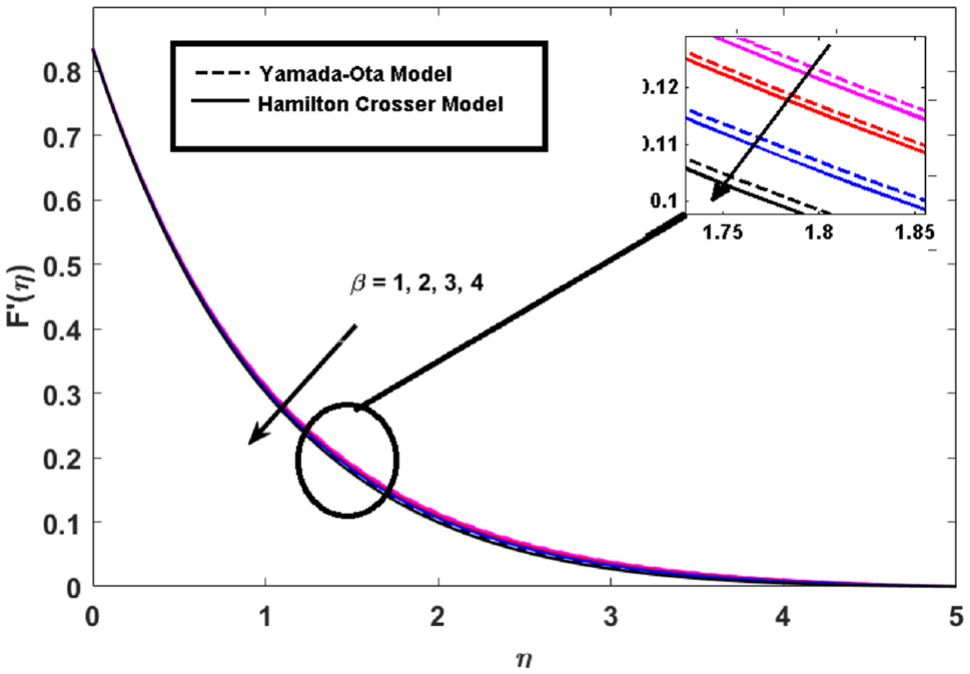
Hydrodynamic interaction ($\beta$) on the velocity profile $F^{\prime}(\eta )$.

**Figure 4:**
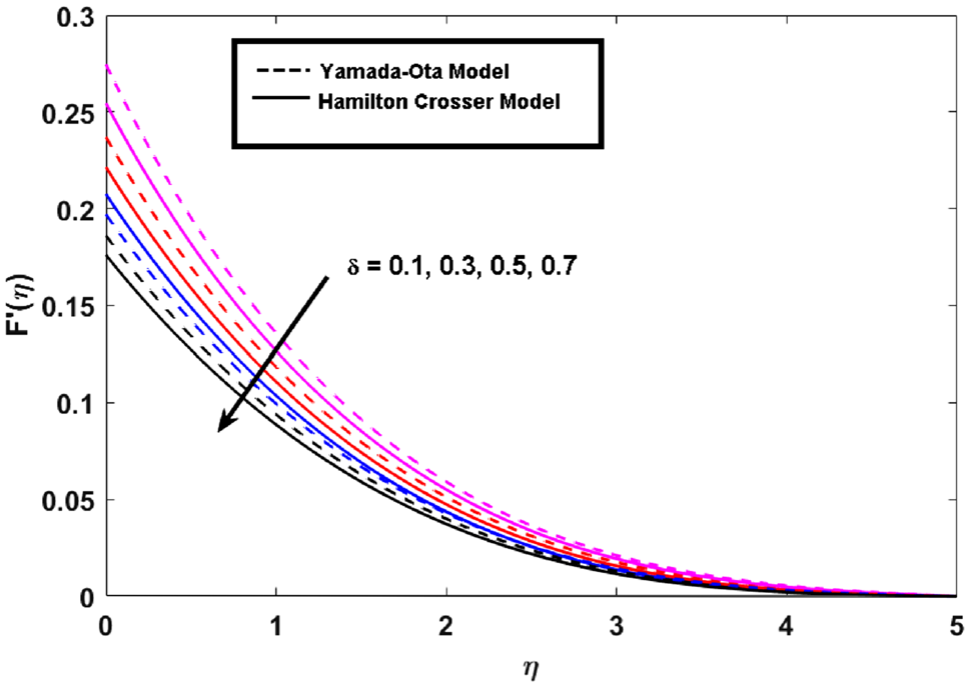
Slip parameter ($\delta$) on the velocity profile $F^{\prime}(\eta )$.

**Figure 5:**
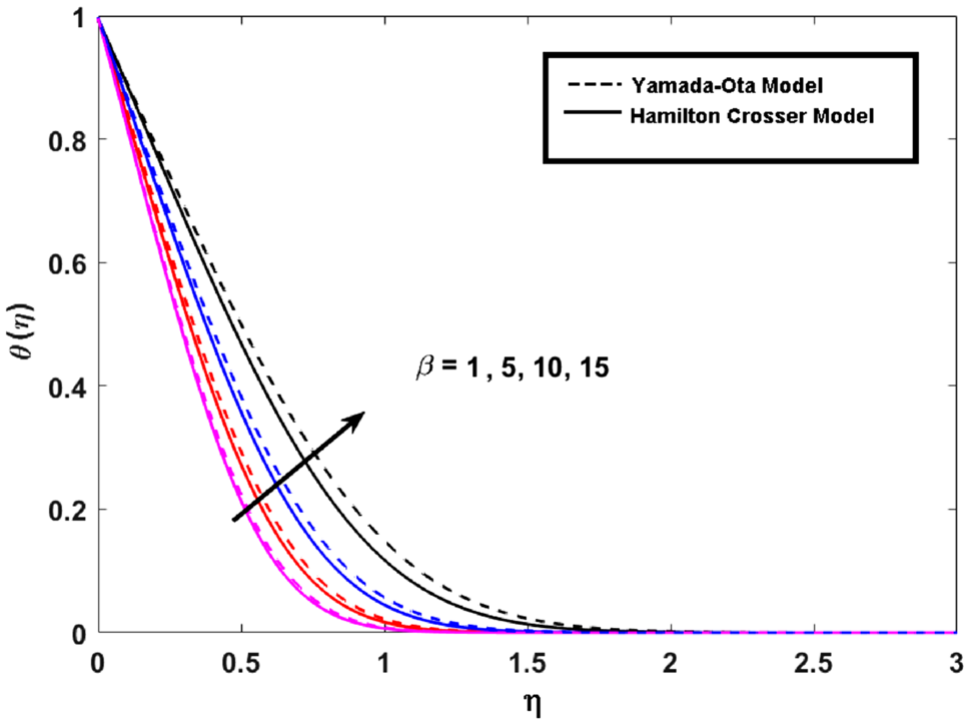
Hydrodynamic interaction ($\beta$) on the thermal profile $\theta (\eta )$.

**Figure 6:**
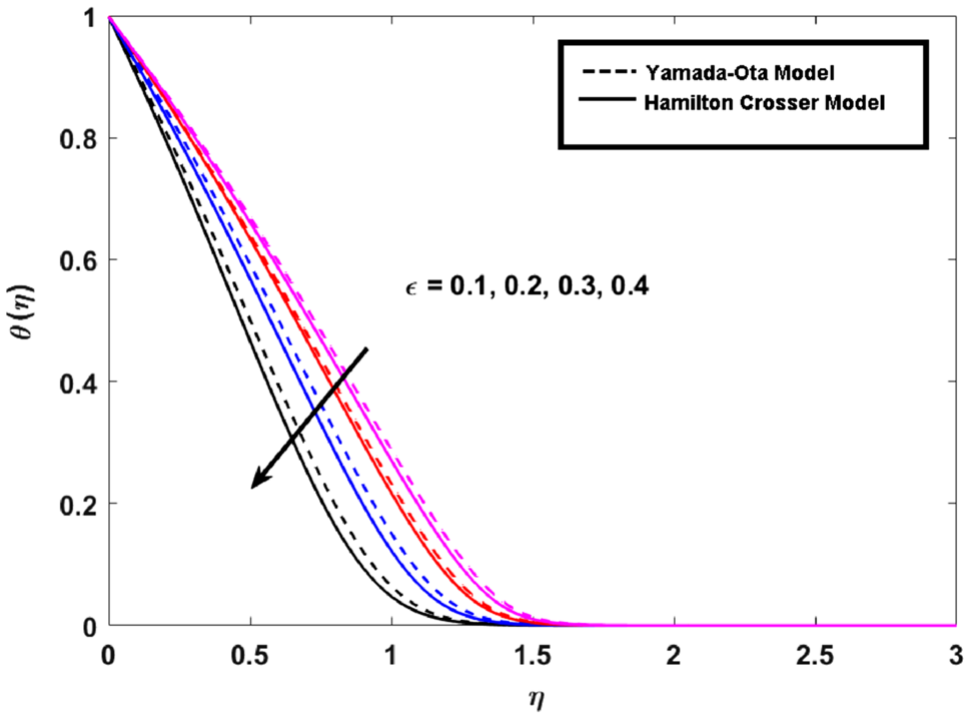
Curie temperature ($\varepsilon$) on the thermal profile $\theta (\eta )$.

**Figure 7:**
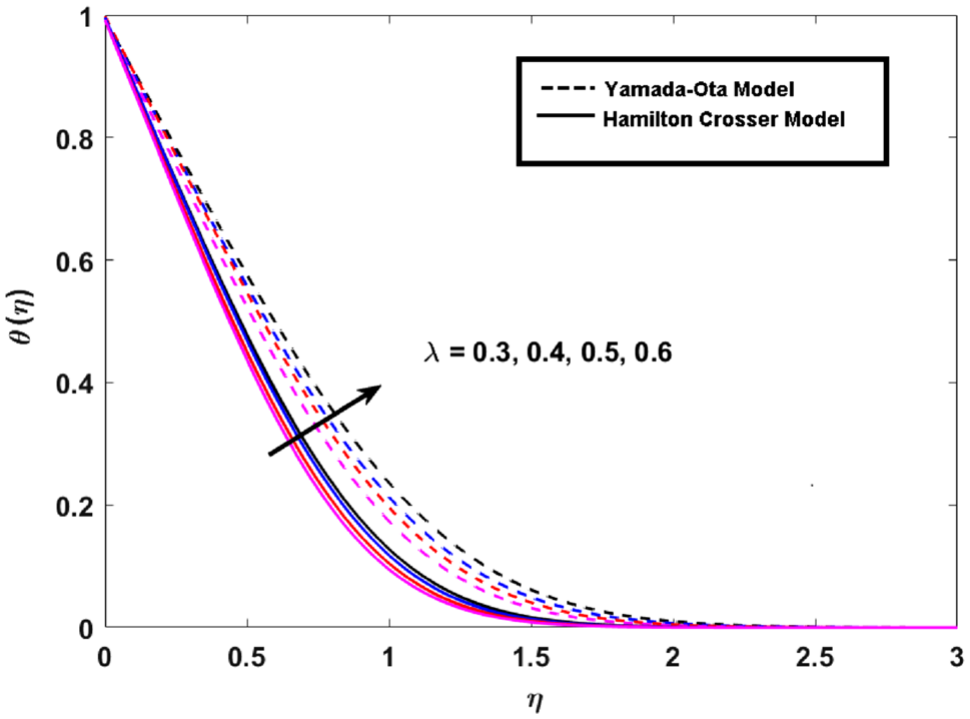
Viscous dissipation ($\lambda$) on the thermal profile $\theta (\eta )$.

**Figure 8:**
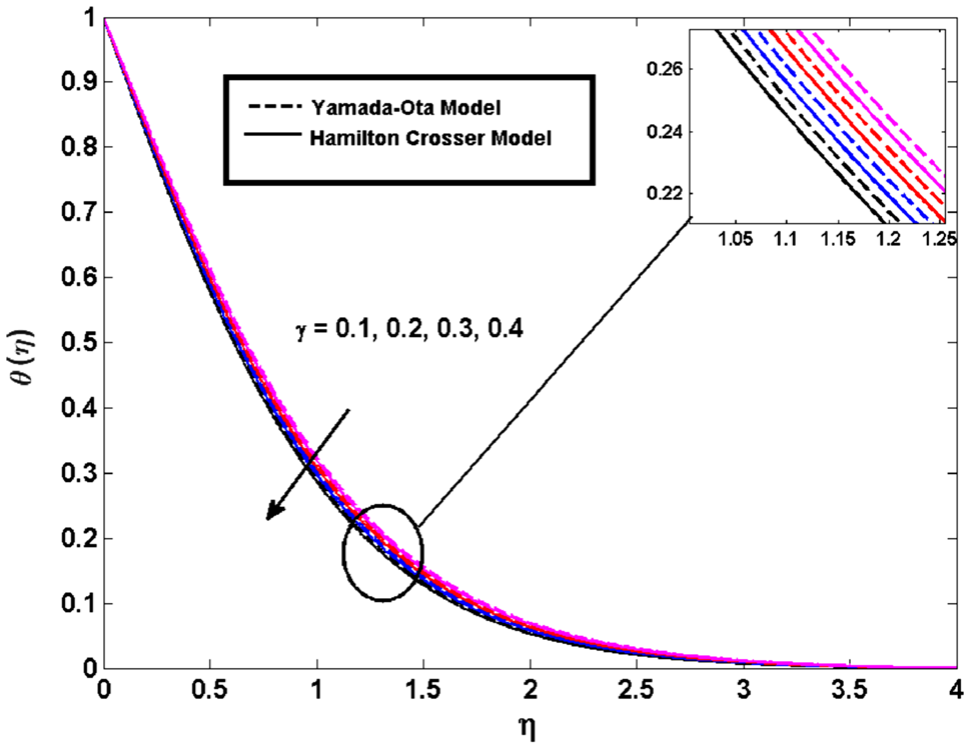
Thermal relaxation parameter ($\gamma$) on the thermal profile $\theta (\eta )$.

**Figure 9:**
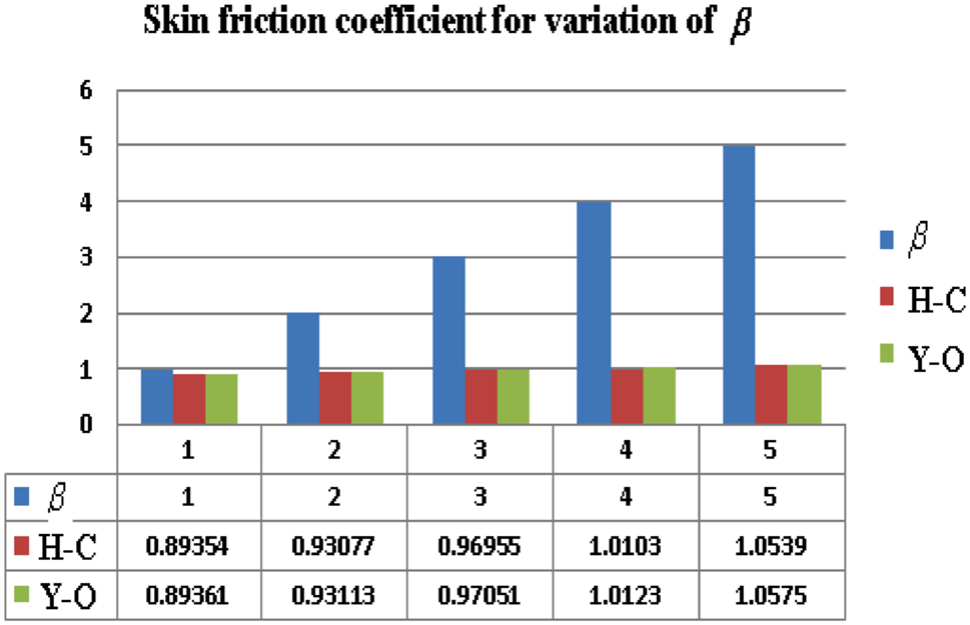
Skin friction $F^{\prime\prime}(0)$ for hydrodynamic interaction parameter $\beta$.

**Figure 10:**
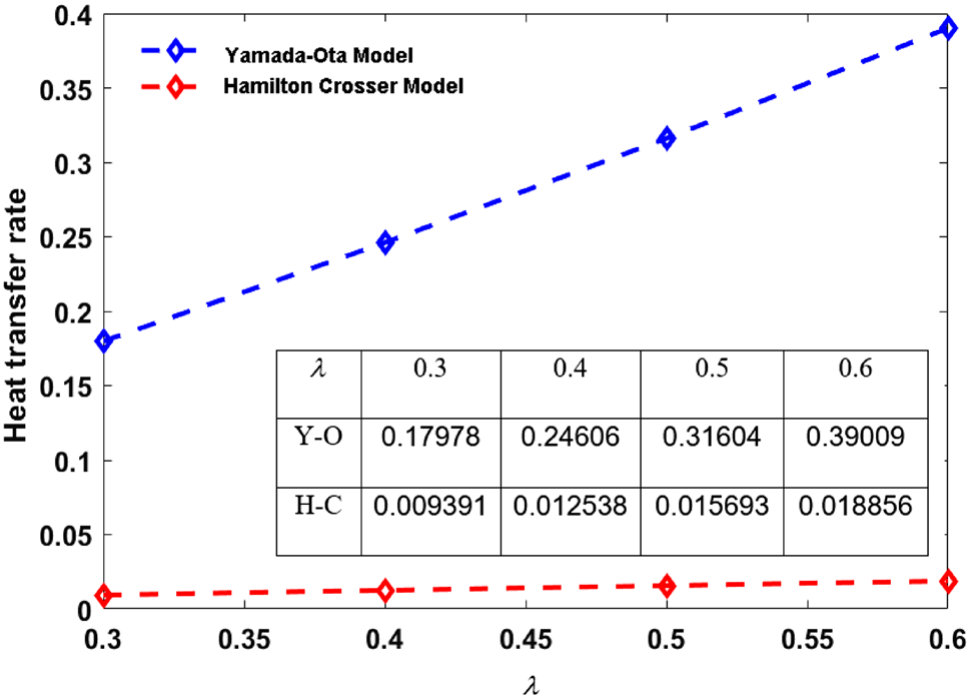
Nusselt number $\theta^{\prime}(0)$ for Viscous dissipation parameter $\lambda$.

**Figure 11:**
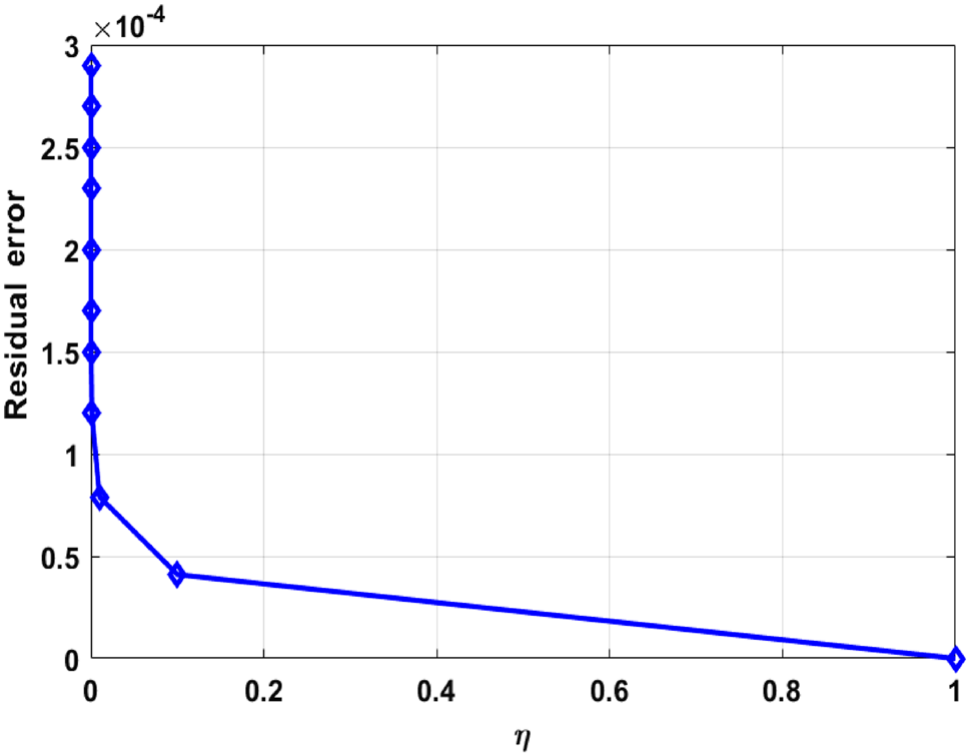
Residual error in the numerical solution by shooting method against $\eta$.

This segment (Figs. 3, 4, 5, 6, 7, 8, 9, 10) is earmarked to witness the influences of the numerous parameters versus associated profiles. The influence of Hydrodynamic interaction $(\beta )$ on the velocity profile is given in Fig. 3. A drop in the fluid axial velocity is seen for $\beta .$ This is because of the presence of nanoparticles that boosts the fluid density and ultimately decrease the fluid axial velocity. The influence of the slip parameter $(\delta )$ on the velocity profile is illustrated in Fig. 4. As the $\delta$ values are raised, the thickness and velocity of the boundary layer decrease. Stretching velocity is transferred to the liquid as the $\delta$ is increased and as a result, the velocity profile decreases. Figure 5 show the outcome of hydrodynamic interaction $\beta$ on thermal profile. It is noted that the thermal profile boost for larger values of $\beta .$ Indeed, when the collision or interaction of molecules of hydrodynamic metals in liquids is increased, the temperature of the liquid rises. It is pertinent to mention here that the impact of the Yamada-Ota hybrid nanofluid model is far ahead of the Hamilton Crosser model. Figure 6 depict the effect of Curie temperature ($\varepsilon$) on thermal profile. It is detected here that thermal profiles decrease for large estimates of $\varepsilon$. This is because the natural magnetic behavior of Curie temperature is lost and as a result, the temperature field diminishes due to the loss of energy. The influence of viscous dissipation parameter ($\lambda$) on thermal profiles is seen in Fig. 7. It is can be seen from the figures that the temperature rises as viscous dissipation rises. Because we know that the fluid viscosity influences its temperature, raising viscous dissipation values boosts the heat of the liquids, causing the liquid temperature to rise. The correlation between the thermal relaxation parameter ($\gamma$) with the thermal energy profile is illustrated in Fig. 8. It is comprehended that the thermal energy profile is decreased for $\gamma {.}$ It takes extra time for material particles to transfer heat to neighboring particles for larger $\gamma .$ In reality, the material has a non-conducting property for large $\gamma ,$ which reduces the fluid temperature. Figure 9 displays the effects of drag force coefficient for the variation of $\beta .$ It is noted that for large estimates of $\beta ,$ drag force is rising. It should be noted that the Yamada–Ota model has a higher drag force than the Xue model. Here, the Yamada–Ota model show higher heat transfer rate in comparison to the Hamilton Crosser model. Figure 10 shows the effects on the Nusselt number $\theta^{\prime}(0)$ for variation of Viscous dissipation parameter ($\lambda$). The behavior of the graph shows that the rate of mass increased rapidly with rising estimates of the $\lambda .$ Here, it is pertinent to reveal that the performance of the Yamada–Ota model is far ahead of the Xue model. By adjusting the other parameters, the residual error graphs are shown in Fig. 11. The error is within acceptable bounds, indicating that our numerical technique provides a highly accurate answer.

Table 4 is designed for the values of Prandtl number ($\Pr$) when compared with Chen^33^ and Ramzan et al.^4^ by keeping the extra parameters’ values zero. An excellent correlation between the values is attained.

**Table 4:** By ignoring the extra parameters, Chen^33^ and Ramzan et al.^4^ compared thermal efficiency for different amplitudes of Prandtl number ($\Pr$).

$\Pr$	Chen^33^	Ramzan et al.^4^	Present
0.71	1.0885	1.088497	1.08850
1.00	1.3333	1.333296	1.33271
3.00	2.5097	2.509689	2.50968
10.00	4.7968	4.796794	4.79679

## Conclusions

In this study, we have presented a comparison between two hybrid nanofluid models namely Hamilton Crosser and Yamada–Ota owing to their heat transfer rates. The assumed hybrid nanofluid comprises silicon carbide and titanium oxide as nanoparticles and diathermic oil as a base fluid. The hybrid nanofluid flow is considered over a stretched surface under the magnetic diploe influence with partial slip condition at the boundary of the surface. The problem is addressed numerically. The significant outcomes of the existing study are appended as under:

- Yamada–Ota model heat transfer result is efficient than the Hamilton–Crosser hybrid nanofluid model.
- The fluid velocity reduces on increasing hydrodynamic interaction and slip parameters.
- The temperature of the fluid reduces by increasing the thermal relaxation parameter.
- Both temperature profiles increase for viscous dissipation and higher hydrodynamic interaction parameters.
- The surface drag coefficient is improved for the hydrodynamic interaction parameter.

## Nomenclature

$$T$$: Temperature
$$M$$: Magnetization effect
$$\mu$$: Dynamic viscosity
$\phi$: Nanoparticle volume fraction
$\gamma$: Thermal relaxation parameter
$$\tau_{w}$$: Shear stress
$T_{w}$: Temperature of wall
$$s_{1}$$: First nanoparticle
$\delta_{1}$: Magnetic field region
c: Magnetic dipole’s displacement
$$S$$: Constant
$$\beta$$: Hydrodynamic interaction
$$\varepsilon$$: Curie temperature
$$\rho$$: Fluid density
$$\nu_{F}$$: Kinematic viscosity
$$U_{w}$$: Stretching velocity
$$\delta$$: Slip parameter
$$k$$: Thermal conductivity
$$T_{c}$$: Curie temperature
$$c_{p}$$: Specific heat
$$HNF$$: Hybrid nanofluid
$$NF$$: Nanofluid
$$F$$: Base fluid
$$s_{2}$$: Second nanoparticle
$\gamma_{1}$: Strength of magnetic dipole
$$\nu_{F}$$: Kinematic viscosity
$K$: Pyro-magnetic coefficient
$$\Pr$$: Prandtl number
$$\lambda$$: Viscous dissipation
$\alpha$: Magnetic field strength
$$C_{F}$$: Skin friction
